# Stuck in the Present: A Human Lack of Ability to Visualise (Different) Needs in the Future May Hamper Timely Implementation of AAL and Supportive Technology

**DOI:** 10.3390/ijerph19116804

**Published:** 2022-06-02

**Authors:** Ulrike Bechtold, Natalie Stauder, Martin Fieder, Harald Wilfing

**Affiliations:** 1 Institute of Technology Assessment, Austrian Academy of Sciences, 1010 Vienna, Austria; 2 Human Ecology Research Group, Department of Evolutionary Anthropology, University of Vienna, 1030 Vienna, Austria; a01268625@unet.univie.ac.at (N.S.); martin.fieder@univie.ac.at (M.F.); harald.wilfing@univie.ac.at (H.W.)

**Keywords:** ageing in place, active assistive living (AAL), assistive technologies, older adults’ surroundings, socio-relational setup, imagining future needs, getting older in a city, demographic change, life course perspective, policy measures for better living

## Abstract

Cities face an evident demographic change, making assistive technologies (AAL) an interesting choice to support older adults to autonomously age in place. Yet, supportive technologies are not as widely spread as one would expect. Hence, we investigate the surroundings of older adults living in Vienna and analyse their “socio relational setup”, considering their social integration and psychophysical state compared to others (health, fitness, activeness, contentedness). Method: Our data included 245 older adults (age: M = 74, SD = 6654) living in their own homes (2018–2020 with different grades of needing support). We calculated univariate and multivariate models regressing the socio-relational setup on the change of routines, technology attitude, mobility aid use, internet use, subjective age, openness to move to an institutional care facility in the future, and other confounding variables. Results: We found a strong correlation between all categories (health, fitness, activeness, contentedness) of older adults comparing themselves to their peers. Among others, they are significantly related to institutional care openness, which implies that participants who felt fitter and more active than their peers were less clear in visualising their future: unpleasant circumstances of ageing are suppressed if the current life circumstances are perceived as good. This is an example of cognitive dissonance.

## 1. Introduction

European and national research funding and technical development initiatives coined the term “Active and Assisted Living” (AAL). AAL embraces technologies and services which shall support older adults living at home autonomously, improve their quality of life and reduce the financial burdens that an ageing society puts on the social health care system [1,2]. AAL is hence predominantly based upon the paradigm “ageing in place” as the most common (and preferred) form for older adults to live: ageing in place shall be supported by ICT-based (information and communication technology) support of mobility, health, inclusion and the organisation of the daily activities of living [3].

The questions we use in this paper are part of a larger study, and the purpose of the study was to examine the quality of life of older adults in Vienna and their technology use. This paper investigates the connection between social relations of older adults (and the way these are estimated by themselves) and their concrete surroundings (ideas about the way they would want their flats), the way they see themselves (subjective age), and their own attitudes towards changes of different sorts (moving to a care facility, changing their environments, e.g., homes and/or routines). All these factors are relevant for older adults’ attitudes towards technologies to support them. These technologies are not as widely spread as those who finance them (policymakers) have hoped, and those who develop (technology developers) and those who sell (companies) these technologies would want.

By focusing on “active ageing” at home as part of “AAL”, the responsibility to age in a successful way is delegated to the individuals. This may obscure the view on infrastructures, interactions, and living conditions in a more holistic way, and policymakers may escape their responsibility to create age-friendly infrastructures on a larger scale.

Additionally, this may overlook the fact that older adults are not a homogenous group, as Van Dyk et al. [4] point out. For this reason, the AAL approach may well lead to missing out or even excluding those older adults who are most in need of support, as Wan et al. [5] exemplify in the case of telemedicine.

Despite a history of more than ten years of research, AAL applications often fail to find their way into the daily lives of older adults. Zhang et al. [6] state, “Despite the potential benefits of AAL solutions, the uptake and widespread deployment is still far from being mature.” Other sources attest to AAL technologies a lack of practical use [7,8,9,10,11,12], and the approach of the triple win (market development, ease of economic burden, and rise of quality of life of older adults) is sometimes questioned [13,14]. It seems essential to investigate the contexts of older adults’ lives as the actual place to implement such technologies [15]. Urban agglomerations—including Vienna—are subject to constant growth and, therefore, will face severe demographic change, expected in terms of a constantly changing age structure [16]. Austria’s population is expected to pass 9 million [17] in 2030, and 25% will be older than 65 [18]. Currently, the more significant part of the over 65-year-old group in Austria live in their own homes [19]. Still, only a small proportion of those living at home have adapted their homes in any way to ease access or increase security and facilitate individual mobility [19]. The capital of Austria, Vienna, expects a significant increase in the older population between 65 and 79 years until 2028, and the count of people older than 80 years is expected to double [20]. Against this background, it seems relevant to have a closer look at the concrete surroundings of older adults living in Vienna, taking their living conditions and their relation to technology, and their view on their own life into focus.

In the literature, a potentially positive connection between social integration and ageing well can be found [21,22,23]. A positive effect of social embeddedness and active participation in community life on older adults’ physical and mental health is mentioned [24,25]. However, according to Heo et al. [26], findings are often conflicting. Bechtold et al. [27] found a strong connection between the quality of life and mobility instead of social integration. Seeing the importance and heterogeneity of the social aspect in the literature, we kept “social integration” as a factor but added another facet. Our research hypothesis is that the interaction with other people may be relevant to older adults’ attitude toward AAL technology not only in a direct way (covered by “social integration”) but also, or even more so, by the way older adults see themselves compared to others in concrete psychophysical aspects.

### Framing of the Research Design

Extending our research question from an actual social relation status as the primary factor to the way older adults see themselves in relation to others, we developed a socio-relational setup, which consists of how older adults firstly are actively integrated into their social surroundings, (e.g., frequency of real contacts) and secondly, how they relate themselves to others in terms of evaluating their psychophysical assets in comparison to others (health, fitness, activeness, and contentedness).

We investigate the interrelation between the socio–relational setup against factors that potentially influence technology adoption. These are flexibility in daily routines, attitude towards a built environment change, and technology use preferences. We are using the variables “social integration” and “attitudes toward ageing” as the socio-relational setup and relate them to the variables “change of routines”, “technology attitude”, “mobility aid use”, “internet use”, “subjective age”, and “institutional care openness”. We add the category of “subjective age” as a control for the relational facet of how older adults see themselves compared to others and as a potentially relevant factor for how people are positioned in the socio-relational setup. Because AAL is related to the goal of people staying at home as long as possible (“ageing in place”), we also investigate the influence of the socio-relational setup in terms of their openness to possibly move to an institution at a later point, if needed.

## 2. Method

The following sections explain the research design, including the relevant aspects of the questionnaire used, the target group, and the privacy and pretesting of the questionnaire.

### 2.1. Research Design and Research Questions

In our research, we used a questionnaire to obtain self-reported measurements on the perception of the participants’ own ageing, their assessment of their environments, and their own attitudes towards changes of different sorts (moving to a care facility, changing their environments, e.g., homes and/or routines). These are reliable and valid tools, according to [28]. We used technology attitude as besides access to technologies and sufficient training, the “attitudes toward technology are important predictors of technology adoption” [12]. More in detail, we refer to Wang et al. ([29], p. 3), who state that such measures capture older adults’ “(…) overall psychosocial situation” from their perspective and hence provide a “better methodological homogeneity and comparability of the condition of groups across studies and countries”.

**Figure 1:**
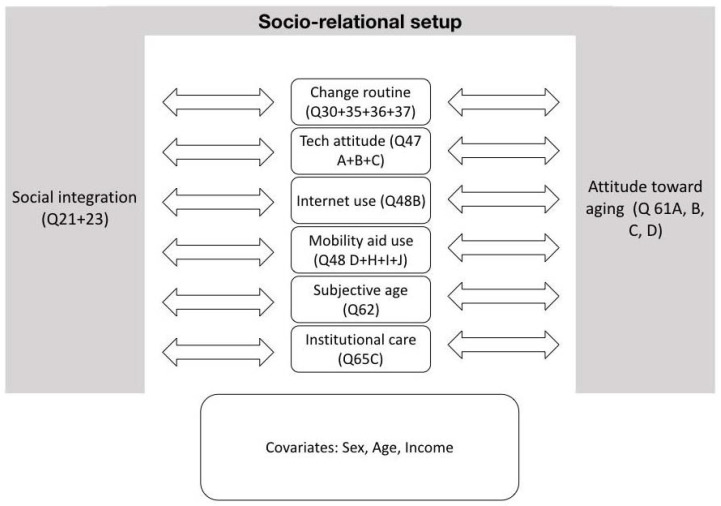
Depicts the socio-relational setup, consisting of a direct (left pillar in grey) and indirect component (right pillar in grey) of older adults as they relate to others. The latter comprises how health, fitness, activeness, and contentedness are rated compared to others, and the former embraces the factual estimation of the social integration of the questioned older adults. This setup is tested against the variables in the centre (as indicated by the arrows) and other confounding variables (sex, age, income).

Based on this, the research questions are whether the socio-relational setup significantly correlates with how flexible people sketch their future. Whether they want to (or already have) implement(ed) changes in their built and surrounding environments, and whether (or not) they can imagine getting older in an institutional setting. We also investigated their technology use patterns, mobility (aid uses), and subjective age, as Figure 1 depicts. We also investigate the correlation between the four variables that make up the right pillar of the socio-relational setup, which embraces the fitness, health, activeness, and contentedness of the questioned people in relation to their peers.

*Socially integrated older adults and socially isolated older adults (Q21 + Q23)* were defined by the questions “Do you have regular contact (at least several times a month) with your most important contact person?” and “Are you in regular contact (at least several times a month) with one or more of your neighbours?” The participants who answered both questions with a “yes” were labelled “socially integrated”, and those who chose “no” to one or both questions were labelled “socially isolated”.

*Older adults’ attitude toward ageing (Q61A, Q61B, Q61C, Q61D)* was defined by the questions “Compared to others my age, I feel…healthier/less healthy/unsure”, “Compared to others, I feel fitter/less fit/unsure”, “Compared to others I feel more active/less active/unsure”, “Compared to others I feel more content/less content/unsure”. The answer “unsure” in all of the above questions was relabelled as a “missing answer” for further statistical analyses.

*Change of routines (Q30 + Q35 + Q36 + Q37)* was defined by the questions “Thinking of your daily paths outside of your home, would you modify anything, if you could?”, “Have you made any changes in your living environment to make your home more comfortable for yourself?”, “Would you want to make changes to your living environment without incurring costs?”, “Do you plan to make any specific changes in your living environment in the near future?” The possible answers were coded as 0 = “no” and 1 = “yes”. We added all numbers up to use them for further statistical analysis. Higher numbers, therefore, indicated a higher interest in changing their own home and living environment.

*The question which defined technology attitude is (Q47A + B + C),* “Would you be prepared to invest in a technological system, and would you change your habits and environment for it to support yourself?”

*Mobility aid use (Q48D + Q48H + Q48I + Q48J)* was defined by the question, “Do you use an escalator, walking aid, rollator, or wheelchair?” Participants who answered “Yes” to at least one of these questions were relabeled as “mobility aid users”.

*Internet use (Q48B)* was defined by the question “Do you use the internet?”

*Subjective age (Q62)* was made up of the two questions “How old are you?” and “How old do you feel?” Participants who indicated that their subjective age was younger than their calendrical age were labelled as “feeling younger”. In comparison, participants who showed an older perceived age than their calendrical age were labelled as “feeling older”

*Institutional care openness (Q65C)* was defined by the question, “To what extent do you agree with the following statement: I can well imagine living in a setting of institutional care.” With the answers “agree”, “somewhat agree”, “somewhat disagree”, and “disagree”. Following the univariate analysis (see Section 3.2), we recategorised the answers based on the results into “pronounced opinion” (formerly “agree” and “disagree”) and “weak opinion” (formerly “somewhat agree” and “somewhat disagree”) for further multivariate analysis (see Section 3.3).

The survey questions represent categorical variables such as sex or variables that indicate an order. In the case of more than two (binomial model), we assume linearity among different levels on the Likert scale, as answers are evenly distributed in most cases. The questionnaire covers the following main aspects (all questions are provided in the previous section).

### 2.2. Target Group

The target group was defined as older adults (65 yrs plus) living in their own homes (with varying levels of support: from no support at all to daily delivery of support or care services). We did systematic desk research (mainly online) on ageing organisations in and around Vienna, which dealt with people living in their own homes. All of the identified organisations were approached by e-mail. Those who responded are mentioned below and were contacted in person, and the authors received a number of invitations to talk at various events to distribute the questionnaire. Moreover, individual organisations and individuals (hospital settings) were contacted. Older adults were approached by the authors predominantly personally.

There were a total of 478 distributed questionnaires. A total of 135 of the 478 questionnaires were distributed without a personal face-to-face explanation beforehand (in institutional settings). However, these people received a group introductory or a written guide. Of the 478 questionnaires distributed, we received 225 completed questionnaires back. Our response rate is 47%. According to Baruch and Holtom [30], an average response rate for individual distribution is 52.7% (SD = 20.4), and an average response rate for organisational distribution is 35.7% (SD = 18.8). Therefore, we assume that our response rate is sufficient, and our sample is, regarding the chosen community, representative.

Several organisations were approached as multipliers (numbers of distributed questionnaires in brackets): Vienna Pensioners Clubs (100), Viennese social services (45), the acute gerontology department of the Viennese Hartmann Hospital of the Elisabeth Hospital group (50); association “alters.kulturen “, Vienna (30), Board of the Austrian Senior Expert Pool in Vienna (58), “information day on care” of the Vienna Chamber of Labour (30), association “culture in Vienna “, Vienna (35), Seniors at WUK, Vienna (30), “Seniors Dance Club “, Vienna (15), Students of the University Vienna, approaching their grandparents and neighbours (35), “Seniors Club “, Graz (20), association “Vita Activa at the Karl Franzens University “, Graz (20), Parish in Vienna and Lower Austria (5), individual older adults, as registered within the database of former participatory projects (5).

### 2.3. Privacy and Pretesting

The questionnaire was anonymous, and the categories were adapted to foster privacy, (e.g., no postal codes were collected, but the size of place of residence was asked), and no lists of people or locations of gathering the questionnaires were kept at any time. The authors explained to the participants the purpose of the study orally, the structure of the questionnaire, and how the data will be used. All this was also provided in written form. In the first test phase of the questionnaire in 2016, 4 out of 15 tested people expressed their unwillingness to address the topics raised in the questionnaire related to deficits of ageing quite openly (two of them were older than 80 years, the other two belonged to the younger cohort between 65 and 70). This led to a refinement of the explanations throughout the questionnaire and a paragraph that entitled the people questioned to omit parts of the questionnaire if they were unwilling to think about “ageing”. Some minor adaptations were made following the pretesting of the wording and structure of the questions and options to answer.

### 2.4. Data Processing

Two hundred and forty-five correctly filled questionnaires were given/sent to the authors and therefore made up the data basis. We used SPSS IBM, Version 27, and R 4.0.5 (Author: R Core Team, Organisation: R foundation for Statistical Computing, Vienna, Austria, 2020).

### 2.5. Univariate and Multivariate Statistics

Together with the survey’s design, we planned the statistical analysis consisting (i) of descriptive statistics and testing of in-between group differences and (ii) our number of cases and thus the power is sufficient [31]. We used multivariate regression models, controlling for sex, and SES (education and income).

We calculated a correlation matrix for the four variables “1. Compared to others my age, 2. I feel healthier/less healthy; fitter/less fit; 3. more active/less active; more content/less content” (Q61 A, B, C, D).

In the *univariate analysis,* we calculated 30 chi-squared tests. We tested the variables

1. “social integration” (Q21 + Q23);
2. “Compared to others my age, I feel healthier/less healthy (Q61A);
3. fitter/less fit (Q61B);
4. more active/less active (Q61C); and
5. more content/less content” (Q61D) separately on the variables;

(a) “Change of Routines” (Q30 + 35 + 36 + 37);
(b) “Technology Attitude” (Q47 A + B + C), “Mobility Aid use” (Q48D + H + I + J);
(c) “Internet use” (Q48B);
(d) “Subjective Age” (Q62);
(e) Institutional Care Openness” (Q65C).

In the *multivariate analysis*, we calculated 24 separate models in total. We regressed the depending variables:

1. “Compared to others my age, I feel healthier/less healthy (Q61A);
2. fitter/less fit (Q61B);
3. more active/less active (Q61C); and
4. more content/less content” (Q61D) separately on the variables;

(a) “Change of Routines” (Q30 + 35 + 36 + 37);
(b) “Technology Attitude” (Q47 A + B + C), “Mobility Aid use” (Q48D + H + I + J);
(c) “Internet use” (Q48B);
(d) “Subjective Age” (Q62);
(e) Institutional Care Openness” (Q65C).

We controlled for years of age, sex, and income as a continuous variable based on a binomial error structure.

## 3. Results

### 3.1. Descriptive Results

In the following, we present a descriptive overview of the sample and the results of some questions that seem relevant to us in the context of our research question, (e.g., attitude towards ageing in place). Although some have not been used for further in-depth analysis, these results are still included here as we think they are essential in light of the contextualisation of our calculated model and the conclusions we draw. Table 1 presents the socioeconomic factors used as covariates in the multivariate statistical analysis.

#### 3.1.1. Ageing in Place and Care

Of the older adults surveyed, 98.7% (226) want to live at home as long as possible (*N* = 229). There was an explicit willingness to make specific adaptations in their home environment in 63.9% or 156 people (*N* = 244). About one-third, or 36.1% (75), could well imagine moving into an institution in the coming years (*N* = 208).

Once care is thought to be needed, 76.5% or 176 people *(N* = 230) tend to choose professional assistance as the right choice instead of care from a family member (32.6% or 75 people; *N* = 230). This is the hypothetical need for care. Of the 34.9% or 82 people (*N* = 235) who actually need and receive care, it is relatives or family members who actually provide the care in 50.6% (40) of the cases (*N* = 79). In line with this, most of the questioned people do not want to burden their family economically (94% or 206 people; *N* = 219) but still prefer to use social benefits (89.3% or 192 persons; *N* = 215).

#### 3.1.2. Home Adaptations and Technology Use

When asked about their willingness to invest money, change their home environment, and change their habits to acquire a technical system that was helpful for them, 38.4% (84) of the participants in our sample showed an interest (*N* = 219).

In our sample, 67,1% (159) of participants used the internet (*N* = 237), with men (75% or 51 people; *N* = 68) using the internet more frequently than women (61.8% or 55 people; *N* = 89).

#### 3.1.3. Subjective Ageing

Nearly all of the questioned people (156, 94.5%) feel younger than they actually are (*N* = 165).

Looking back, getting older is neither bound to a particular birthday (78.6% or 169 people; *N* = 215) nor a specific event (59.9% or 121 people; *N* = 202) for the questioned people.

### 3.2. Univariate Results

All categories of our socio-relational setup in terms of older adults comparing themselves to others correlate significantly with each other (see Table 2).

#### 3.2.1. Social Integration, Change Routines, Technology Attitude

No statistically significant relationship was found between “social integration” and any tested variables. As the variable “social integration” (Q21 + Q23) was not statistically significant for any of the variables in the chi-squared tests, we excluded it from further multivariate analysis. Further, no significant relationships were found between the variables “change routines,” “technology attitude,” and “comparison with others”; (see Table 3).

#### 3.2.2. Institutional Care Openness

The relation between fitness compared to others and institutional care openness was significant, X^2^ (3, *N* = 203) = 18.279, *p* = 0.000. Participants who felt fitter than their peers were more likely to either somewhat agree or somewhat disagree with being open to moving to a care facility at a later point in time. Participants who felt less fit than their peers were more likely to (strongly) agree or (strongly) disagree with being open to moving to a care facility.

The relation between activeness compared to others and institutional care openness was significant, X^2^ (3, *N* = 205) = 23.382, *p* = 0.000. Participants who felt more active than their peers were more likely to either somewhat agree or somewhat disagree with being open to moving to a care facility at a later point in time. Participants who felt less active than their peers were more likely to (strongly) agree or (strongly) disagree with being open to moving to a care facility (see Table 3).

### 3.3. Multivariate Results

We found that all “Health”, “Fitness”, “Activeness”, and “Contentedness” regress significantly positively on “Mobility aid use”. “Fitness”, “Activeness”, and “Contentedness” regress significantly negatively on “Internet use”; both “Activeness” and “Contentedness” regress significantly negatively on “Subjective age”; and both “Fitness” and “Activeness” regress significantly on “Institutional care openness”. We did not find any significant association with “Change of routines” or “Technology attitude” (see Table 4). There was also no significant association with the controlling variables (data not shown).

## 4. Discussion

Our results show that the socio-relational setup is one-sided in our sample. How older adults are actively integrated into their social surroundings, (e.g., frequency of real contacts) was not significant with any other variables. Our study did not find any association between social integration and older adults’ attitude toward their own health and contentedness. However, the part that asks how they relate themselves to others in evaluating their own psychophysical assets compared to their peers (health, fitness, activeness, and contentedness) is highly significant in many aspects, which will be discussed in the following. These findings are contrary to other research on the topic [32], though it must be noted that research in this field of interest is likely to show mixed findings [26].

*Subjective Age.* The vast majority of respondents feel younger than they are (94.5%), which is in line with Rubin and Berntsen [33], who state that from the age of 25 onwards, we tend to perceive ourselves as younger than we are. Our results show a connection between older subjective age and a more negative estimation of life satisfaction and fitness compared to others, which is in line with the literature. An older subjective age than calendrical age has been linked to being at future risk of poorer memory and cognitive function in older adults [34,35]. These findings are also in line with Hubley et al. [36], who found that older subjective age is typically linked to poorer health variables. Weiss and Lang [37] see the prevalence of negative age stereotypes as reinforcing the discrepancy in peoples’ perception of their own age.

Van Orden [38] discusses the idea that older subjective age could be linked to internalised ageism, which could explain our findings that older adults who stated a subjectively older age had also estimated themselves to be less content than their peers. The way older adults perceive their ageing in our sample is in line with a life course perspective: looking back, getting older is neither bound to a particular birthday (78.6%) nor to a certain event (59.9%) for the majority of the questioned people.

Additionally, from an anthropological point of view, a certain unwillingness of older people to deal with their own age and ageing can be connected with negative images of old age and the tabooing of old age in our society. The phenomenon of the acceleration of the present, e.g., through media “flooding,” has also been discussed for some time [39] and seems relevant in the light of societally addressing age-related issues.

The focus on ageing is often directed towards the end of life, reinforcing the tendency to repress it. However, a (collective) repression mechanism is also understandable because it is biologically expectable that a period at the end of life will be associated with decrepitude and a need for help (see also [40]).

*Visualising future institutionalised care.* We found that participants who felt fitter and more active than their peers were less clear in visualising their future. They were significantly more likely to have a weak opinion on future care needs than those who felt less active and less fit than their peers, who expressed a significantly more pronounced opinion on possibly moving to a care facility at a later point in their life. Fleuren et al. [41] found that having a shorter subjective remaining life expectancy (<25 years) led older adults to be more likely to engage in advance care planning. This indicates that being confronted with actual psychophysical challenges leads to a clearer idea of how people want to be cared for when they get old.

In addition, from a life course perspective, Carstensen [42] builds her socioemotional selectivity theory on the fact that getting older changes the priorities whether or not we want to confront ourselves with more difficult aspects of life, (e.g., planning of old age), and this could be related to the factual experience of difficulties (see also [43]).

The fact that our results show that a positive valuation of one’s socio-relational setup (that is, a good state of fitness and activeness as compared to others) makes people more indifferent to more concrete ideas of how they want to be cared for in the future and aligns well with the theory of cognitive dissonance, which originated in social psychology [44]. The unpleasant circumstances of ageing, which are undoubtedly present and discussed in society, are perceived as dissonance with the self-assessment of life circumstances that are currently perceived as good. The cognitive contradiction is resolved by actively repressing the (possible but not yet experienced) unfavourable circumstances of ageing and thus resolving the dissonance.

Another theoretical approach that is interesting in the light of these results originates from the field of environmental science [45] is the “value-action gap” (also known as “attitude-behaviour gap”, “intention-behaviour gap”, “believe-behaviour gap or knowledge-attitudes-practice gap”). It describes the phenomenon that surveys show severe discrepancies between attitudes and how they are expressed by the participants and the actual patterns of action the same individual pursues. For example, in environmental science, it is clearly measurable that attitudes toward sustainable consumption are much more pronounced than actual purchasing behaviour shows [46]. The “value-action gap” can be seen in our results: most participants have, would, or would like to change their environments. Still, only those who have clear ideas about their own possible future care needs, who already perceive a more difficult everyday life, (e.g., they feel less fit and less active than their peers). In addition, staying at home as long as possible is an explicit wish of nearly all questioned people (98.7%). Yet, as soon as care is thought to be needed, 76.5% tend to choose professional support over care provided by a family member (32.6%). This result can be another example of the “value-action gap” mentioned above. Yet, it affects the hypothetical need for care when the wish to remain at home is not necessarily connected to being cared for by a family member.

When it comes to an actual care situation (a good third of the questioned people who need and receive care), for more than half of them (50.6%), this care is administered by relatives or family members. In line with this wish to not be dependent on a family member, most of the people questioned do not want to burden their family economically (94%) but instead go for social support (89.3%).

Although 98.7% of the people questioned want to live at home as long as possible, only 63.9% show an explicit willingness to make specific adaptations in their home environment. This indicates a certain lack of imagining physical changes that have not yet occurred. Our results could not find a significant correlation between how prepared people were to initiate changes to improve their personal living environment and how they felt compared to their peers.

*Internet use.* Heo et al. [26] found a correlation between internet use and well-being and social integration. However, we do not see the latter association. Our results show that self-perception (in the categories of activeness, fitness, and contentedness) in comparison to others is significantly related to internet use and mobility aid use. That means that more (virtual) integration and more mobility connect to better rating themselves as opposed to others.

This is also in line with Nakagomi et al. [47], who found a positive correlation between cognitive and physical fitness and internet use, and Wan et al. [5], who found that subjective health was a robust predictor of internet use. Although our health category is not significant here, our other categories of fitness, activeness, and contentedness are significant. Additionally, Poli et al. [48] state that older adults who showed symptoms of poorer physical health were less likely to partake in digital health research than other peers their age. Within our four questions (health, fitness, activeness, contentedness), contentedness was the most significant aspect in relation to internet use. Contentedness can probably be seen as an indicator that those participants, who have found good ways to deal with potentially existing issues in health, activeness, and fitness, are more likely to use the internet. Nakagomi et al. [47] and Chen and Persson [49] state that for older adults, the use of the internet is an indication of well-being and cannot, like for the younger generation, be generalised as an indication of adverse psychological effects.

Our sample has a high preparedness and willingness to use ICT (information and communication technology), and 67.1% use the internet regularly. This exceeds the results König et al. [50] found, who state that 48% of the Austrian population, older than 50 years, uses the Internet regularly. In 2020 EU-wide, 61% of the older adults (aged 65–74) used the internet in the last three months, whereas the differences for the member states are quite large (Croatia has 28% as opposed to Denmark with 94%, Austria displays 58%) [51]. Based on the SHARE database, Halmdienst and Schmidt [52] found that people who use(ed) computers in their professional careers are more likely to display digital literacy. Additionally, a clear gap between lower and higher socioeconomic levels was shown. Our sample is characterised by a socioeconomic profile above the Austrian average, so the higher internet use rate is not surprising.

*Mobility aids.* Participants who felt healthier, more active, fitter, and more content than their peers were less likely to use (need) mobility aids. While the value of self-rated health as a means of evaluating actual health and mortality is backed up by a body of research [53,54,55], it is a noteworthy finding that our four categories of health, fitness, activeness, and contentedness compared to peers also seems to be a good predictor of actual health conditions.

## 5. Conclusions

In this paper, we take a closer look at the surroundings of older adults living in the city of Vienna, taking their living conditions, social integration, built environment, and relation to technology and their view on their own lives into focus. We do so by implementing what we call the socio-relational setup, which consists of a direct and indirect component to be in relation to “the others”. The latter comprises how health, fitness, activeness, and contentedness are rated compared to others, and the former embraces the factual estimation of the social integration of the questioned older adults. This setup is tested against the change of routines, technology attitude, mobility aid use, internet use, subjective age, and openness to move to an institutional care facility in the future. Our results were not significant in terms of social integration, but our four categories, health, fitness, activeness, and contentedness compared to peers, seem to be a good predictor of actual health conditions. This seems worthwhile to take into consideration for future research.

Looking at our findings in the light of a life course perspective, one could say that the most notable insight is that it is more difficult for older adults to imagine a future situation and visualise the process of getting older when they perceive themselves more positively (compared to others) and feel younger than they actually are.

This seeming lack of prospective imaginative capacity is consistent with cognitive dissonance and the so-called “value-action gap” that explains the difference between what we think are desirable (future) actions and how we actually decide to behave (in the future).

To actually bring AAL (active and assisted living) and other supportive technologies into the lives of older adults means that the healthcare sector or marketing strategies can only, in minimal ways, rely on older adults’ capacities to imagine themselves into a (possibly different) future. So, a possible future need for AAL is a weak argument to improve their own homes with AAL and other supporting technologies. This presents an even more significant challenge, as perceived age is likely to be more important than actual age in deciding to introduce AAL.

The fact that older adults who need AAL the most may experience the most significant barriers to accessing certain AAL services implies simplifying the welfare and support landscape for older adults in Austria to make the Austrian health care system more accessible for senior citizens. Such a simplification could facilitate access to helpful information, support, and orientation, (e.g., technical support opportunities, care allowance) in AAL applications. Moreover, strategies to implement AAL in peoples’ lives on a larger scale need more deliberate structural thought and governance (policy measures). These could also build upon existing arenas and platforms that embrace all areas of older adults’ lives, e.g., senior clubs and all sorts of older adults’ organisations and digital initiatives. The latter could be promising for the integration of older adults as our sample showed that those who proactively used the internet also displayed a more positive rating as to their fitness, activeness, and contentedness compared to others.

In addition, our findings underline a connection between a positive self-perception of older adults and some proactive strategies, (e.g., use of mobility aids, attitude towards institutional care). This correlation is often emphasised by Gerontechnolgists and other experts (of the practice) and makes another challenge visible: the current societal perception of the topic “ageing” and the process of getting requires a joint attempt to overcome the so far predominantly negative framing of ageing in public and the media.

Relying on this, the strategy to sell AAL along with biographical age may not serve its purpose. The question of how it is possible to better align AAL directly with the needs and problems actually felt by its users and thus defuse them better is not a question of a specific time that we can easily predict. The aim needs to be to clarify what technologies can mean within the process of ageing and what role the immediate social and spatial environment and information environment can play in this process.

A different starting point may be needed for policymakers who allocate resources and strategic structural planners of urban environments and neighbourhoods. It is not about finding THE right moment to successfully approach an older adult because that may either not exist (due to successfully suppressing the process of getting older), or these moments are too diverse in terms of quality and timing to be easily identified. Our results show a seeming lack of prospective imaginative capacity as long as age-related issues have not yet occurred, which can be explained by cognitive dissonance and the “value-action gap”.

Therefore, a different strategy than to offload the problem of active ageing onto the individual, onto older adults’ backs, is needed. It could be far more promising to invest more thought, money, and energy in the direction of structural changes, which are based on thinking of lifetimes rather than moments in life. Such changes could provide fundamental advantages for users of all ages. Additionally, they could contribute to a change in attitudes rather than forcing individuals at certain (necessarily arbitrary) points of their lives to take preventive action and implement inconvenient changes in their living environments. However, such a structural approach faces the challenge of carefully balancing a culture of individuality and freedom with regulations to implement age-friendly surroundings.

Yet, as the same logic of cognitive dissonance to suppress not yet acute challenges is necessarily also true for policymakers, they may not be inclined to worry about issues unless they become massive and their solving would promise immediate success. That is why it is essential to consciously and commonly think about strategies, decisions, and interventions in an inclusive way to achieve the changes needed, even without them being connected to an immediate reward. As human beings, we are all stuck in the moment, hence thinking about the future requires deliberate effort.

## Figures and Tables

**Table 1:** Summarises the socioeconomic factors.

**Age**	**Mean (in yrs.)**	**SD (in yrs.)**
	74	6654
**Sex**	**Frequency**	**Percent**
Male	91	37.3%
Female	151	61.6%
**Income**		
Below 500 Euro	7	2.9%
500–1000 Euro	24	10%
1000–2000 Euro	93	38.9%
2000–3000 Euro	58	24.3%
3000–4000 Euro	20	8.4%
4000–5000 Euro	8	3.3%
More than 5000 Euro	8	3.3%

**Table 2:** Displays the correlation matrix showing the Pearson correlation coefficients and significances; correlation is significant (indicated by **) at the 0.01 level (2-tailed).

	Health Compared to Others (61A)	Fitness Compared to Others (61B)	Activeness Compared to Others (61C)	Contentedness Compared to Others (61D)
Health compared to others (61A)	1			
Fitness compared to others (61B)	0.580 **	1		
Activeness compared to others (61C)	0.553 **	0.562 **	1	
Contentedness compared to others (61D)	0.444 **	0.402 **	0.446 **	1

**Table 3:** Shows the chi-squared tests including estimates and significances (* *p* < 0.05; ** *p* < 0.01; *** *p* < 0.001) of the variables “Social Integration”, “Health”, “Fitness”, “Activeness” and “Contentedness” tested against “Change of routines”, Technology Attitude”, “Internet use”, “Mobility aid use”, “Subjective age”, and “Institutional care openness”.

	Change Routines (Q30 + Q35 + Q36 + Q37)	Technology Attitude (Q47)	Mobility Aid Use (Q48D + H + I + J)	Internet Use (Q48B)	Subjective Age (Q62)	Institutional Care Openness (Q65C)
Social Integration (Q21 + Q23)	4.149	0.479	2.736	0.905	1.458	7.703
Health compared to others (Q61A)	4.249	0.015	21.260 ***	5.175 *	11.866 ***	6.501
Fitness compared to others (Q61B)	5.060	1.664	28.581 ***	6.094 *	11.811 ***	18.279 ***
Activeness compared to others (Q61C)	7.740	1.059	15.037 ***	15.394 ***	10.536 **	23.382 ***
Contentedness compared to others (Q61D)	1.309	0.686	10.142 **	3.893	5.489 *	4.419

**Table 4:** Regression matrix, estimates, and significances (* *p* < 0.05; ** *p* < 0.01; *** *p* < 0.001) of the regression of “Health”, “Fitness”, “Activeness” and “Contentedness” on “Change of routines”, “Subjective age”, “Mobility aid use”, “Internet use”, “Technology attitude” and “Institutional care openness”, controlling for sex, age, education and income (not shown).

	Change Routines (Q30 + Q35 + Q36 + Q37)	Technology Attitude (Q47)	Mobility Aid Use (Q48D + H + I + J)	Internet Use (Q48B)	Subjective Age (Q62)	Institutional Care Openness (Q65C)
Health compared to others (Q61A)	−0.138	0.070	1.958 ***	−0.583	−18.03	−0.676
Fitness compared to others (Q61B)	0.123	−0.326	2.068 ***	−0.896 *	−17.272	1.486 ***
Activeness compared to others (Q61C)	−0.101	−0.339	1.586 ***	−0.9134 *	−2.681 *	1.336 ***
Contentedness compared to others (Q61D)	−0.213	−0.393	1.419 ***	−1.053 **	−1.738 *	0.534

## Data Availability

Not applicable.
